# Editorial: Emerging physical implementation for neuromorphic computing: Recent advances and future challenges

**DOI:** 10.3389/fnins.2022.1041997

**Published:** 2022-09-27

**Authors:** Chunsheng Jiang, Qilin Hua, Hai Jiang

**Affiliations:** ^1^Key Laboratory of Integrated Circuits and Microsystems, Education Department of Guangxi Zhuang Autonomous Region, School of Integrated Circuits, Guangxi Normal University, Guilin, China; ^2^Beijing Institute of Nanoenergy and Nanosystems, Chinese Academy of Sciences, Beijing, China; ^3^Design for Reliability, HiSilicon, Chengdu, China

**Keywords:** artificial intelligence (AL), neuromorphic computing, hardware implementation, emerging devices, energy-efficiency

## Introduction

Nowadays, artificial intelligence (Al) chips are widely used in our daily life, such as facial recognition, text recognition, and natural language processing. Especially, low-power Al chips are an urgent need for edge computing. The current commercial Al chips are almost entirely accelerators for deep learning algorithms based on the traditional von Neumann architecture. However, such Al chips suffer serious challenges of tremendous power consumption, high latency, and low area-efficiency on account of the physical separation of processing units and memory.

In recent years, neuromorphic computing paradigms inspired by the biological brain can be implemented by hardware electronics (e.g., CMOS circuits or emerging nanodevices) and have attracted much attention from global researchers, attributed to their potential advantages of high energy-efficiency, massive parallelism, in-memory computing, and high area-efficiency. Nevertheless, neuromorphic systems still face some technical challenges before they could be in mass production. This issue aims to deepen our fundamental understanding of the fabrication process, device physics, architecture, algorithms, and large-scale neuromorphic systems.

## Device physics and design

The basic modules of neuromorphic computing chips are artificial synapses and spiking neurons. Emerging nano-devices are developed as artificial synapses and neurons to achieve lower power dissipation, higher integration density, and lower latency in replace of CMOS circuits. Some kinds of emerging nano-devices have been developed to emulate the functionalities of synapses and neurons. Among them, novel devices (e.g., memristors, and PCRAM) can work as artificial synapses, and the others (e.g., threshold switching and spin-torque device) can usually be used as artificial neurons.

However, the emerging nano-devices inevitably exhibit some non-ideal characteristics, such as nonlinearity, small G_max_/G_min_ ratio, and large device-to-device variation, which limit the large-scale integration of artificial neuromorphic devices. Two kinds of optimization strategies are proposed to address this issue. The one is to weaken non-ideal effects by optimizing the device design and fabrication process. The other is to develop fault-tolerant training/learning algorithms, which can accommodate the imperfections and unreliability of the hardware. Shen et al. proposed a kind of novel gate tunable RRAM structure by adding a MOS structure. The electrical characteristics can be modulated by its back-gate bias. A physics-based stochastic simulation is leveraged to investigate the device physics of the gate tunable RRAMs. This study demonstrated that filament evolution can be controlled by the back-gate bias. As a result, the nonlinearity can be reduced and the G_max_/G_min_ ratio can be improved when a negative back-gate bias is given. In addition, a neural network for handwritten digit classification is constructed based on the gate tunable RRAMs. The simulation shows the accuracy of the neural network is improved when a negative back-gate voltage bias is given, which confirms the superiority of this optimized device structure.

The working mechanisms of most emerging neuromorphic devices are not clear enough until now, especially for artificial neurons. NbO_x_-based threshold switching (TS) is one of the top candidates which can build leaky integrate-and-fire (LIF) spiking neurons. Ding et al. developed an empirical model of on/off resistance of NbO_x_-based TS calibrated with the experimental data. The underlying physics of the LIF neuron circuit are systematically studied by leveraging the developed model. It is demonstrated that both spiking frequency and energy consumption per spike can be optimized by altering the input voltage bias and device parameters. A spiking neural network (SNN) is constructed to control the cart-pole using a reinforcement learning algorithm, which demonstrates that the NbOx-based LIF neuron has great potential in practical applications.

## Computing architecture and system

SNN and coupled-oscillatory network (ONN) are two kinds of dominating architectures for the neuromorphic systems due to their rich dynamics. Even though CMOS-based hardware implementation has lower energy-efficiency and area-efficiency than that consisting of emerging beyond-CMOS devices, the current large-scale neuromorphic system is primarily realized by the commercial CMOS process because of its maturity. Versatility and high integration density of neuromorphic systems are major goals in the future. Pehle et al. present the detailed system architecture and practical applications of the second generation of the BrainScale neuromorphic architecture called as BrainScale-2. Compared with the original BrainScale system, a rapid on-chip calibration method is introduced to enable the scalability of the unit of scale, which improves the integration density of BrainScale-2. Besides, the hybrid plasticity is achieved by combining the analog core with two embedded plasticity and control processors, which makes the system flexible and configurable. As a result, the versatility of BrainScale-2 is improved. The authors demonstrate that the BrainScale-2 system has great potential in many practical applications such as emulating multi-compartmental neuron models.

ONN was proposed as a kind of emerging non-von Neumann architecture in recent years. Especially, the oscillators show oscillation behavior in parallel and encode information by the phase, which results in lower power dissipation and fast computing speed in ONN. Abernot et al. report a small-scale (up to 60 oscillators) proof-of-concept of the ONN computing paradigm fully accomplished by an FPGA-based digital circuit. For the first time, the task of digits recognition is performed efficiently based on hardware implemention of ONN. It validates that the ONN can be used to build a more extensive neuromorphic system and has great potential for edge computing.

## Emerging applications

Currently, Al chips not only have made great achievements in the traditional fields (e.g., computer vision), but also show the growing potential in some emerging applications, such as unmanned vehicle systems and intelligent diagnosis. To realize the automatic analysis of electrocardiogram (ECG), Kang et al. propose a beat-level interpretation method based on the automatic annotation algorithm and object detector. A cascade RCNN is constructed to perform the automatic heartbeats annotation and heartbeat classification, and it achieves high classification accuracy. Even though this algorithm is deployed on the traditional ANN in this paper, it's not difficult to deploy it on SNN with the well-known ANN-to-SNN method.

## Conclusion

Neuromorphic computing paradigms are considered as one of the most promising candidates for next-generation AI systems. Endeavors from device design to system architectures must be made to boot the versatility and increased integration density, before they can be commercially available for emerging applications. This Research Topic shows some latest and most remarkable progress in this field, and we sincerely hope that readers can get some inspiration from this issue to advance their own research and promote the development of bio-realistic artificial intelligence era.

## Author contributions

All authors listed have made a substantial, direct, and intellectual contribution to the work and approved it for publication.

## Funding

This work was supported in part by the National Science Foundation of China (Nos. 61904164 and 61904012) and the Guangxi Science and Technology Base and Talent Special Project (Grant No. AD22035213).

## Conflict of interest

HJ was employed by Design for Reliability, HiSilicon. The remaining authors declare that the research was conducted in the absence of any commercial or financial relationships that could be construed as a potential conflict of interest.

## Publisher's note

All claims expressed in this article are solely those of the authors and do not necessarily represent those of their affiliated organizations, or those of the publisher, the editors and the reviewers. Any product that may be evaluated in this article, or claim that may be made by its manufacturer, is not guaranteed or endorsed by the publisher.

